# *Pneumocystis jirovecii* pneumonia in developing countries*

**DOI:** 10.1051/parasite/2011183219

**Published:** 2011-08-15

**Authors:** Y. De Armas Rodríguez, G. Wissmann, A.L. Müller, M.A. Pederiva, M.C. Brum, R.L. Brackmann, V. Capó De Paz, E.J. Calderón

**Affiliations:** 1 Pathology Department, Institute of Tropical Medicine “Pedro Kourí” Ciudad de la Habana Cuba; 2 Pneumocystis Study Group, Infectology Unit, Hospital de Clínicas de Porto Alegre, Federal University of Rio Grande do Sul Porto Alegre Brazil; 3 Centro de Investigación Biomédica en Red en Epidemiología y Salud Publica, Virgen del Rocío University Hospital Sevilla Spain

**Keywords:** *Pneumocystis*, HIV, opportunistic infection, developing countries, *Pneumocystis*, VIH, infection opportuniste, pays en développement

## Abstract

*Pneumocystis* pneumonia (PcP) is a serious fungal infection among immunocompromised patients. In developed countries, the epidemiology and clinical spectrum of PcP have been clearly defined and well documented. However, in most developing countries, relatively little is known about the prevalence of pneumocystosis. Several articles covering African, Asian and American countries were reviewed in the present study. PcP was identified as a frequent opportunistic infection in AIDS patients from different geographic regions. A trend to an increasing rate of PcP was apparent in developing countries from 2002 to 2010.

## Abstract (fr)

La pneumonie due à *Pneumocystis jirovecii* (PcP) est une infection mycosique sévère chez les patients immunodéprimés. Dans les pays développés, les données épidémiologiques et cliniques de la PcP sont bien documentées. En revanche, dans les pays en voie de développement, on dispose de peu d’informations concernant la prévalence de la pneumocystose. De nombreux articles qui concernent des pays d’Afrique, d’Asie et d’Amérique sont passés en revue dans ce travail. La PcP est une infection opportuniste fréquente chez les patients atteints de sida dans différentes régions géographiques. Une tendance à l’augmentation de l’incidence de la PcP a été observée dans les pays en développement entre 2002 et 2010.

## Introduction

Pneumonia caused by *Pneumocystis jirovecii* (PcP) (previously known as *P. carinii*) has long been recognized in patients with impaired immunity. It was initially described as a cause of epidemic interstitial pneumonia in premature and malnourished infants. Until 1980, PcP was uncommon and recognized in patients who were immunocompromised because of malignancies, immunosuppressive therapy, or congenital immunodeficiencies. However, the rate of infection by *P. jirovecii* increased with the emergence of the human immunodeficiency virus (HIV) (Calderón *et al.*, 2002).

Despite a decline in the incidence of PcP in the era of highly active antiretroviral therapy (HAART), it remains a common and serious opportunistic disease in HIV-infected individuals. In developed countries, the epidemiology and clinical spectrum of PcP have been clearly defined and well documented. In contrast, a limited number of epidemiological studies have evaluated PcP prevalence in developing countries (Morris *et al.*, 2004).

Fisk and colleagues previously reviewed changes in PcP rates among HIV patients in Africa, Asia, India, the Philippines, and in Central and South America. They found a greater percentage of PcP was described compared to the results of earlier studies, indicating that PcP is a significant AIDS-related opportunistic infection (OI) in many developing countries (Fisk *et al.*, 2003).

Recent reports have described an increased rate of PcP in Africa, Asia and South America (Worodria *et al.*, 2003; Aderaye *et al.*, 2007; Le Minor *et al.*, 2008; Panizo *et al.*, 2008; Vray *et al.*, 2008). In this study, we review published studies that have reported the frequency of PcP in developing countries, focusing mainly on more recent data.

### PcP in Africa

PcP was initially thought to be a rare manifestation of AIDS in Africa (Elvin *et al.*, 1989). In Uganda, pneumocystosis was not detected among AIDS patients (Serwadda *et al.*, 1989). Also, while a study of HIVinfected black adults in South Africa found that only one (0.6%) out of 181 patients had PcP (Karstaedt, 1992). On the other hand, rates of 3.6 to 11% were reported for Tanzania, Congo and Ivory Coast in the first decade of the AIDS epidemic (Carme *et al.*, 1991; Lucas *et al.*, 1991; Abouya *et al.*, 1992; Atzori *et al.*, 1993).

Other studies, however, have demonstrated higher rates of PcP in populations on the African continent. In Zimbabwe, PcP was identified by methenamine silver staining in bronchoalveolar lavage samples in 33% of 64 patients with respiratory symptoms who were sputum smear-negative for acid-fast bacilli (AFB) (Malin *et al.*, 1995). In Kenya, *P. jirovecii* was detected by immunofluorescence and toluidine blue staining in respectively 37.2 and 27.4% of 51 HIV/AIDS patients with bilateral pulmonary shadows who were sputum smear-negative for AFB (Chakaya *et al.*, 2003). In a study of African miners in South Africa, PcP incidence at the time of autopsy increased progressively from 9/1,000 in 1996 to 66/1,000 in 2000 (Wong *et al.*, 2006). Also, in an Ethiopian population, PcP was detected by polymerase chain reaction in 42.7% of 131 HIV-infected patients with atypical chest X-ray findings and whose sputum was smear-negative for AFB (Aderaye *et al.*, 2008).

*P. jirovecii* is a common cause of pneumonia in HIVinfected children ages 3-6 months but is less common and less severe in children over age 12 months (Chintu *et al.*, 2002, Jeena *et al.*, 2005). In African children, PcP is an opportunistic pneumonia that is frequently related to HIV infection, and several reports have revealed high rates of PcP in this population. In Zimbabwe, lung biopsies from 24 HIV-seropositive children who died of pneumonia in 1995 were examined in an autopsy study using histology, culture, microscopy and polymerase chain reaction, and *Pneumocystis* was detected in 16 (67%) children (Nathoo *et al.*, 2001). In another study of autopsies carried out in Ivory Coast, PcP was the cause of death in 31% of children under 15 months of age infected with HIV (Lucas *et al.*, 1996). In addition, 31% of all deaths and 48% of infant deaths ≤ one year, according to an autopsy study of HIV-positive children in Botswana, were caused by pneumocystosis (Ansari *et al.*, 2003).

Detection of *P. jirovecii* has been reported in clinical specimens collected by noninvasive methods in Africa. Cysts were identified in the induced sputum and nasopharyngeal aspirates in 51 of 105 (48.6%) children using immunofluorescence staining (Ruffini *et al*, 2002) and *P. jirovecii* DNA was detected by PCR amplification in 15 of 22 (68%) oropharyngeal mouth washes from children who died from AIDS-related PcP (Lishimpi *et al.*, 2002).

### PcP in Asia

In Thailand, fewer than 100 cases of PcP per year were described before 1992. However, there was a marked increase in the incidence of cases reported to the Thai Ministry of Public Health, which peaked at 6,255 cases per year in 2000 (Sritangratanakul *et al.*, 2004). In a retrospective study, PcP was diagnosed in 18.7% of 286 HIV/AIDS patients (Anekthananon *et al.*, 2004). In a prospective study, tuberculosis (TB) was the most common diagnosis (44%), followed by PcP (25.4%) and bacterial pneumonia (20.3%) in 59 HIV/AIDS patients with interstitial infiltrates on chest radiographs (Tansuphasawadikul *et al.*, 2005). Further diagnosis of PcP using a noninvasive method (*e.g.* induced sputum) was documented by polymerase chain reaction in 21% of 52 HIV/AIDS patients suspected of PcP (Jaijakul *et al.*, 2005). In addition, there was a high mortality among patients with acute respiratory failure caused by PcP in Thailand (Boonsarngsuk *et al.*, 2009).

In Cambodia, the HIV/AIDS epidemic has become a major issue in recent years (Bendick *et al.*, 2002). In one study, a total of 381 cases of HIV-infected patients admitted to a public hospital in Phnom Penh between 1999 and 2000 were reviewed, and chronic diarrhea was the most frequent (41.2%) HIV-related problem, whereas PcP was identified in only 8.4% of the patients (Senya *et al.*, 2003). Also, studies have documented low rates of PcP in Vietnam (Louie *et al.*, 2004; Klotz *et al.*, 2007). More recently, PcP was detected in 52-55% of HIV-infected patients with smear-negative sputum for AFB in Cambodia and Vietnam, suggesting that this pneumonia might be a major concern in this region (Le Minor *et al.*, 2008).

Several studies conducted in India have shown a low number of PcP cases, with rates of 5-6.1% described in some reports (Lanjewar *et al.*, 2001; Kumarasamy *et al.*, 2003; Rajagopalan *et al.*, 2009). On the other hand, PcP was found to be an AIDS-defining illness with significant mortality among HIV-infected patients in the HAART era in another report (Kumarasamy *et al.*, 2010). Improved detection of *P. jirovecii* using PCR in several respiratory specimens from HIV-infected and non-infected Indian patients with lung infiltrates and clinical features of PcP has been described, with sensitivity and specificity of 100% and 99% for PCR, and 30.7% and 100% for microscopy by Gomori methenamine silver staining (Gupta *et al.*, 2007). Nested- PCR for the gene encoding the large-subunit rRNA (mtLSUrRNA) also has proved to be a very sensitive tool for *P. jirovecii* detection (Gupta *et al.*, 2009).

### PcP in the Americas

#### • Mexico, Central America and the Caribbean islands

Little is known about *Pneumocystis* infection in Central America. To date, few studies on PcP have been reported for this region. In Mexico, one of the first studies was conducted at four hospitals between March 1984 and January 1989, and *Pneumocystis* infection was diagnosed in 24% of 177 AIDS patients. Cytomegalovirus (69%) and TB (25%) were the most common infections (Mohar *et al.*, 1992). Similar rates of PcP were found in an autopsy study of 58 AIDS patients (Jessurun *et al.*, 1990).

The first case of AIDS in Panama was confirmed in 1985. Ten years later, pulmonary pneumocystosis was diagnosed by bronchoalveolar analysis in 46% of HIV-infected patients with respiratory symptoms (Rodríguez *et al.*, 1996).

Research evaluating PcP in Guatemala found that 52 HIV-infected patients admitted to the Adult Outpatient Clinic of the San Juan de Dios General Hospital had opportunistic infections (OIs), and 14 (27%) of them were diagnosed with PcP. The results of this clinical study, performed between January 1991 and June 1992, suggest that limitations in the diagnostic and laboratory facilities of the hospital hindered the identification of some OIs (Estrada *et al.*, 1992).

The first cases of PcP in Cuba were reported in 1969 (Rodriguez-Vigil *et al.*, 1969). *Pneumocystis* infection was described in malnourished children, indicating that professionals must be aware of PcP while treating this population in health services in developing countries (Razón *et al.*, 1977). A PcP rate of 45% was described in 40 HIV-infected Cuban patients based on clinical signs, symptoms and chest radiographs (Menéndez Capote *et al*, 1992). An autopsy study carried out in the same country showed pneumocystosis in 32% of 211 HIV patients with severe immunosuppression (Arteaga *et al.*, 1998).

In Barbados, PcP was diagnosed in 37.8% of 47 children also diagnosed with HIV between 1981 and 1995. This OI was the most common (65.2%) cause of death in this population with a mortality rate that was higher among those patients diagnosed in infancy compared to those diagnosed in post-infancy (Kumar *et al*, 2000; St John *et al.*, 2003).

In a study of a Haitian population, Pitchenik and colleagues found pneumocystosis in 35% of AIDS patients (Pitchenik *et al.*, 1983). On the other hand, only two patients with PcP among 29 AIDS patients were reported from another study in Haiti (Malebranche *et al.*, 1983).

The clinical spectrum of the AIDS epidemic was described for Puerto Rico, and the three main diagnoses for AIDS, as reported by the local AIDS Surveillance Program from 1981 to 1999, were wasting syndrome (30.7%), esophageal, bronchial and pulmonary candidiasis (29.4%) and PcP (26.8%) (Gomez *et al.*, 2000). Of the 377 pediatric AIDS cases reported between 1981 and 1998 on the island, PcP accounted for 23% and was the most common AIDS-defining condition in the referred population (Perez-Perdomo *et al.*, 1999). Moreover, PcP affected 49 out of 100 HIV adult patients who died from AIDS in this region between 1982 and 1991 (Climent *et al.*, 1994). In addition, a high prevalence of PcP was observed in 1,308 HIVpositive injection-drug users in Puerto Rico from 1992 to 2005 (Baez Feliciano *et al.*, 2008).

#### • South America

Several reports have been published in Venezuela, Brazil and Chile (Panizo *et al.*, 2008; Soeiro *et al.*, 2008). Also, investigations have been conducted in Argentina and Peru (Bava *et al.*, 2002; Eza *et al.*, 2006).

The epidemiology of pneumocystosis was reviewed in Venezuela, and PcP was found in 36.6% of HIVinfected patients with respiratory symptoms treated between 2001 and 2006, also suggesting that PcP must be suspected in other populations (*e.g.* cancer patients) with signs and symptoms of lower respiratory tract infection (Panizo *et al.*, 2008).

In 2002, Argentina had the sixth largest number of cumulative pediatric cases of AIDS in the Americas. Therefore, unsurprisingly, PcP had a significant influence on their health services. A study of 389 children at risk for or infected with HIV-1 conducted from February 1990 to June 1997 found that severe bacterial infection and PcP were the most common AIDS-defining conditions and death-related diseases among AIDS patients (Fallo *et al.*, 2002). Another study demonstrated a high rate of PcP (35%) among AIDS patients in intensive care units (Bava *et al.*, 2002). Although the comparison of the pre and post-HAART era in Argentine AIDS patients revealed an increased PcP rates from 5.9% to 9.4%, TB was the main cause of hospital admission for HIV patients in both study periods (Pérez *et al.*, 2005).

The first cases of infection by *Pneumocystis* in Chile were described in 1960 in patients with interstitial plasma cell pneumonias (Bustamante *et al.*, 1960). Later, PcP was the most common lung disease in HIV-infected patients, causing 37.7% of respiratory episodes in 236 assessed patients (Chernilo *et al.*, 2005).

AIDS is a significant public health problem in Brazil, with 362,364 cases reported as of June 2004 and an estimated total of over 600,000 HIV-infected adults at that time (Wissmann *et al.*, 2006). In one study, among 35 HIV-positive patients with respiratory symptoms, PcP was the most frequently identified AIDS-related disease (55%) followed by TB (41%) (Weinberg *et al.*, 1993). Another study detected pneumocystosis in 27% of 250 HIV/AIDS patients who died from respiratory acute failure in São Paulo between 1990 and 2000 (Soeiro *et al.*, 2008). The introduction of HAART in Brazil led to a significant change in the AIDS scenario. A total of 2,821 cases were assessed in 18 Brazilian cities, with TB (26%) being more frequent than PcP (14%) as AIDS-defining condition, possibly in part because of the anti-PcP prophylaxis used (Marins *et al.*, 2003).

The use of newer and more potent immunosuppressive agents has been associated with PcP unrelated to AIDS in developed countries (Sepkowitz *et al.*, 2002). Radisic and colleagues observed 17 cases of PcP in kidney transplant recipients in Argentina between July 1994 and July 2000 (Radisic *et al.*, 2003) and more studies focusing on this point are necessary in developing areas.

## Discussion

In the present study, we reviewed several articles that described *Pneumocystis* infection in developing countries (Table I). Significant rates of PcP have been reported that show *P. jirovecii* as a pathogen that causes a common OI in AIDS patients in many countries.

**Table I.** *Pneumocystis* pneumonia in developing countries.

Country (Years)	Patient population	PcP patients/total (%)	PcP diagnosis	HAART coverage rate *	PcP chemoprophylaxis	PcP mortality rates	Reference
**Africa**							
Uganda (1999-2000)	HIV/AFB smear negative with respiratory symptoms	32/83 (39)	DFA on BAL	33%	25%	NA	Worodria *et al.*, 2003
Kenya (1999-2000)	HIV/AFB smear negative with respiratory symptoms	19/51 (37)	TBS and DFA on BAL	38%	All patients within five days prior or after the bronchoscopy procedure	26.3%	Chakaya *et al.*, 2003
Ethiopia (2004-2005)	HIV/AFB smear negative with respiratory symptoms	39/131 (30)	DFA on sputum and BAL	29%	NA	NA	Aderaye *et al.*, 2007
Ethiopia (2004-2005)	HIV/AFB smear negative with respiratory disease	56/131 (43)	PCR on sputum and BAL	29%	NA	NA	Aderaye *et al.*, 2008
Botswana (1997-1998)	AIDS	11/35 (31)	Histopath, H & E and GS stains	79%	NA	28.6%	Ansari *et al.*, 2003
Zambia (1997-2000)	HIV dying with respiratory disease	52/180 (29)	Histopath, H & E and MS stains	46%	NA	29%	Chintu *et al.*, 2002
Zambia (NA)	AIDS dying with respiratory disease	15/22 (68)	PCR on OMW	46%	NA	68%	Lishimpi *et al.*, 2002
Senegal (2002-2005)	HIV/AFB smear negative with respiratory symptoms	135/317 (43)	DFA on IS and BAL	56%	19%	19%	Vray *et al.*, 2008
Central African Republic (2002-2005)	HIV/AFB smear negative with respiratory symptoms	135/317 (43)	DFA on IS and BAL	21%	40%	16%	Vray *et al.*, 2008
Malawi (2002-2004)	HIV with respiratory symptoms	6/660 (1)	DFA and real time PCR on IS	35%	NA	NA	van Oosterhout *et al.*, 2007

**Asia (1)**							
Thailand (2000-2006)	HIV with respiratory symptoms	8/14 (57)	DFA and Giemsa stain on BAL and TBBx	61%	7%	64%	Boonsarngsuk *et al.*, 2009
Thailand (2002)	HIV/AIDS without respiratory symptoms	53/286 (19)	Clinical diagnosis	61%	28.2%	NA	Anekthananon *et al.*, 2004
Thailand (2002-2003)	HIV/AIDS with respiratory symptoms	15/59 (25)	Clinical diagnosis	61%	NA	NA	Tansuphasawadikul *et al.*, 2005
Thailand (NA)	HIV/AIDS with respiratory symptoms	11/52 (21)	Giemsa stain and PCR on IS	61%	NA	NA	Jaijakul *et al.*, 2005
Cambodia (1999-2000)	AIDS without respiratory symptoms	32/381 (8)	CXR finding and exclusion of other common causes of pneumonia	67%	100%**	NA	Senya *et al.*, 2003
Cambodia (2002-2004)	HIV/AFB smear negative with respiratory symptoms	84/160 (53)	DFA on BAL	67%	39%	23%	Le Minor *et al.*, 2008
Cambodia (NA)	HIV/AIDS without respiratory symptoms	20/101 (20)	Clinical diagnosis	67%	NA	NA	Bendick *et al.*, 2002
Vietnam (2000)	HIV without respiratory symptoms	5/100 (5)	DFA on IS	26%	4%	20%	Louie *et al.*, 2004
Vietnam (2002-2004)	HIV/AFB smear negative with respiratory symptoms	38/69 (55)	DFA on BAL.	26%	7%	4%	Le Minor *et al.*, 2008

**Asia (2)**							
India (1996-2000)	HIV without respiratory symptoms	36/594 (6)	Standard clinical definitions and by laboratory procedures	NA	NA	28.8%	Kumarasamy *et al.*, 2003
India (1996-2008)	HIV without respiratory symptoms	11/51 (22)	Assess clinical	NA	NA	22%	Kumarasamy *et al.*, 2010
India (2004-2006)	HIV without respiratory symptoms	5/100 (5)	Assess clinical and laboratory findings	NA	100%		2% Rajagopalan *et al.*, 2009

**North America**							
Mexico (1984-1989)	HIV without respiratory symptoms AIDS	43/177 (24)	Histopath, H & E and GS stains	57%	NA	24%	Mohar *et al.*, 1992

**Central America**							
Panama (1995)	HIV with respiratory symptoms	25/55 (46)	MS	56%	NA	NA	Rodriguez *et al.*, 1996
Guatemala (1991-1992)	HIV without respiratory symptoms	14/52 (27)	Clinically	37%	NA	NA	Estrada *et al.*, 1992

**Caribbean Islands**							
Cuba (1986-1995)	AIDS	30/93 (32)	Histopath, H & E and GS stains	> 95%.	NA	4.5%	Arteaga *et al.*, 1998
Cuba (1988-1989)	HIV without respiratory symptoms	18/40 (45)	Clinically and radiologically	> 95%.	NA	NA	Menendez-Capote *et al.*, 1992
Barbados (1981-1995)	HIV without respiratory symptoms	18/47 (38)	Clinically	NA	NA	65.2%	Kumar *et al*, 2000
Haiti (1980-1982)	AIDS without respiratory symptoms	7/20 (35)	Histopath or by TBBx	41%	NA	28%	Pitchenik *et al.*, 1983
Puerto Rico (1992-2005)	HIV without respiratory symptoms	20/143 (14)	Clinically	NA	NA	NA	Báez-Feliciano *et al.*, 2008

**South America**							
Venezuela (2001-2006)	AIDS with respiratory symptoms	15/41 (37)	DFA on sputum, induced sputum and BAL	NA	NA	NA	Panizo *et al.*, 2008
Peru (1999-2004)	HIV/AIDS without respiratory symptoms	2/16 (13)	Histopath, H & E and GS stains	48%	NA	12.5%	Eza *et al.*, 2006
Argentina (1990-1997)	HIV without respiratory symptoms	79/226 (35)	Clinical status	73%	NA	21.6%	Fallo *et al.*, 2002
Argentina (1995-1996) and (2000-2001)	HIV without respiratory symptoms	22/233 (9)	Clinical status	73%	NA	NA	Pérez *et al.*, 2005
Chile (1999-2003)	HIV with respiratory disease	89/236 (38)	Histopath, stains and PCR	82%	18%	22%	Chernilo *et al.*, 2005
Brazil (1990-2000)	HIV/AIDS dying respiratory disease	68/250 (27)	Histopath, H & E and GS stains	80%.	NA	27%	Soeiro *et al.*, 2008

Notes: AFB, acid fast bacilli; DFA, direct fluorescent antibody test; BAL, bronchoalveolar lavage; NA, not available; IS, induced sputum; TBS, Toluidine Blue Stain; H & E, hematoxylin and eosin stain; MS, methenamine silver stains; GS, Grocott silver stain; OMW, oropharyngeal mouth wash; CXR, chest X-rays; TBBx, transbronchial biopsy.

* Global total available at WHO/UNAIDS/UNICEF, Towards Universal Access: Scaling Up Priority HIV/AIDS Interventions in the Health Sector, Progress Report, September 2009: http://www.who.int/hiv/pub/2009progressreport/en/. Country totals available at WHO/UNAIDS/UNICEF, Towards Universal Access: Scaling Up Priority HIV/AIDS Interventions in the Health Sector, Progress Report, June 2008: http://www.who. int/hiv/pub/2008progressreport/en/index.html.

** Information available after hospital admission, no data previously.

Notes: DFA, direct fluorescent antibody test; BAL, bronchoalveolar lavage; NA, not available; H & E, hematoxylin and eosin stain; MS, methenamine silver stains; GS, Grocott silver stain; TBBx, transbronchial biopsy.

* Global total available at WHO/UNAIDS/UNICEF, Towards Universal Access: Scaling Up Priority HIV/AIDS Interventions in the Health Sector, Progress Report, September 2009: http://www.who.int/hiv/pub/2009progressreport/en/. Country totals available at WHO/UNAIDS/UNICEF, Towards Universal Access: Scaling Up Priority HIV/AIDS Interventions in the Health Sector, Progress Report, June 2008: http://www.who.int/hiv/pub/2008progressreport/en/index.html

**Fig 1.**
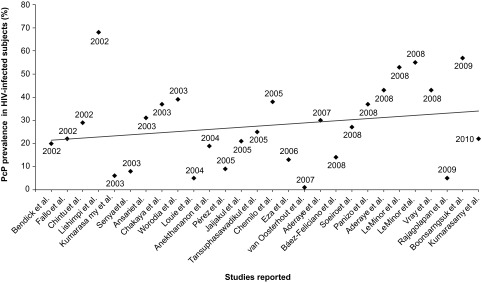
*Pneumocystis jirovecii* pneumonia (PcP) rates among HIV-infected subjects in developing countries from 2002 until 2010.

Fisk and colleagues reviewed this topic previously and showed a trend to an increasing rate of PcP in Africa from 1986 to 2000 (Fisk *et al.*, 2003). We obtained similar data from different HIV populations in African, Asian and American countries, with a linear trend of increasing rates apparent from 2002 to 2010 (Fig. 1).

The percentage of PcP in the HIV/AIDS population has varied among studies, however. Differences in study design, including heterogeneity in the patient population and the diverse laboratory methods used, may explain some of the variability. In this regard, the diagnosis of PcP in low-resource countries is usually clinical (Graham *et al.*, 2001). One study found that the differential diagnosis of pneumocystosis was made by 77% of physicians on the basis of symptoms, chest radiographs and arterial blood gas analyses (Curtis *et al.*, 1995). PcP was commonly unsuspected prior to death because clinicians misdiagnosed it as TB or bacterial pneumonia (Wong *et al.*, 2006).

*Mycobacterium tuberculosis* is the major pathogen identified in almost all studies in developing countries. TB is frequently observed in HIV/AIDS patients with > 200 CD4^+^ cells/mm3 and might lead to death at an earlier stage of HIV infection. PcP rarely occurs with CD4^+^ cell counts > 100 mm^3^. Thus, PcP is an important diagnostic consideration among the most immunocompromised patients (van Oosterhout *et al.*, 2007).

A hypothesis regarding reduced exposure to *P. jirovecii* in developing countries had been previously suggested (Malin *et al.*, 1995), however, antibodies against *Pneumocystis* have been found in 70% of Gambian children (Wakefield *et al.*, 1990) and DNA was detected in specimens from 45 (51.7%) of 87 infants who died in the Chilean community (Vargas *et al.*, 2005).

Miller and colleagues suggested that different *P. jirovecii* genotypes may have distinct physical requirements for survival in the environment and/or for transmission (Miller *et al.*, 2007). Moreover, differences in strains may play an important role in the pathogenesis of this infection. In Africa, genotype 3 according to the mtLSUrRNA sequence was the most frequently obtained (Miller *et al.*, 2003). Recently, the association between genetic polymorphisms of several loci and the intensity of infection from HIV-positive patients was investigated in Europe. In that study, genotype 1 at the mtLSUrRNA was associated with less virulent cases of PcP (Esteves *et al.*, 2010). New studies must be conducted to genotype *P. jirovecii* isolates from HIV patients in developing countries.

In conclusion, significant rates of PcP have been described in HIV-infected patients in the developing world. A trend to increasing rate is also evident from recent African, Asian and American studies. Finally, clinicians must bear in mind that *P. jirovecii* is very important in the differential diagnosis of OIs in the context of developing countries.
